# Ganglioside SSEA-4 in Ewing sarcoma marks a tumor cell population with aggressive features and is a potential cell-surface immune target

**DOI:** 10.1038/s41598-024-62849-8

**Published:** 2024-05-24

**Authors:** Silke Jamitzky, Bianca Altvater, Carolin Krekeler, Laura Hoen, Caroline Brandes, Julia Ebbinghaus, Lisa Richter, Lisa Kosel, Laurin Ochs, Nicole Farwick, Katja Urban, Lena Kluge, Lara Bücker, Dennis Görlich, Ian C. D. Johnston, Rita Pfeifer, Wolfgang Hartmann, Claudia Rossig, Sareetha Kailayangiri

**Affiliations:** 1 Department of Pediatric Hematology and Oncology, University Children’s Hospital Muenster, Albert-Schweitzer Campus 1, 38149 Muenster, Germany; 2 Institute of Biostatistics and Clinical Research, University of Muenster, Schmeddingstr. 56, 48149 Muenster, Germany; 3 Miltenyi Biotec B.V. & Co. KG, Friedrich-Ebert-Straße 68, 51429 Bergisch Gladbach, Germany; 4 Gerhard-Domagk-Institute of Pathology, University of Muenster, Domagkstr. 17, 48149 Muenster, Germany; 5 Cells-in-Motion Cluster of Excellence (EXC 1003 - CiM), University of Muenster, Roentgenstr. 16, 48149 Muenster, Germany; 6 Princess Máxima Center for Pediatric Oncology, Heidelberglaan 25, 3584 CS Utrecht, The Netherlands

**Keywords:** Cancer, Cell biology, Immunology

## Abstract

Carbohydrate markers of immature cells during prenatal human development can be aberrantly expressed in cancers and deserve evaluation as immune targets. A candidate target in Ewing sarcoma is the globo-series ganglioside stage-specific embryonic antigen-4 (SSEA-4). We detected SSEA-4 expression on the cell surface of all of 14 EwS cell lines and in 21 of 31 (68%) primary EwS tumor biopsies. Among paired subpopulations of tumor cells with low versus high SSEA-4 expression, SSEA-4^high^ expression was significantly and consistently associated with functional characteristics of tumor aggressiveness, including higher cell proliferation, colony formation, chemoresistance and propensity to migrate. SSEA-4^low^ versus SSEA-4^high^ expression was not related to expression levels of the EWSR1-FLI1 fusion transcript or markers of epithelial/mesenchymal plasticity. SSEA-4^low^ cells selected from bulk populations regained higher SSEA-4 expression in vitro and during in vivo tumor growth in a murine xenograft model. T cells engineered to express SSEA-4-specific chimeric antigen receptors (CARs) specifically interacted with SSEA-4 positive EwS cells and exerted effective antigen-specific tumor cell lysis in vitro. In conclusion, with its stable expression and functional significance in EwS, SSEA-4 is an attractive therapeutic immune target in this cancer that deserves further evaluation for clinical translation.

## Introduction

Ewing sarcoma (EwS) is an aggressive bone and soft tissue cancer arising from mesenchymal stem cells and molecularly characterized by an aberrant fusion between transcription factor EWSR1 and an ETS family member^1^. Intensive multimodal treatment regimens can cure only a proportion of patients^2^, and outcome is especially poor in metastatic disease^3^. Cellular immune therapy with T cells engineered to express chimeric antigen receptors (CAR) to target cell surface antigens has emerged as a highly effective novel treatment modality in B cell-derived cancers^4–6^. Application of CAR T cell therapy in solid cancers, including EwS, is limited by the lack of adequate target antigens with consistent and homogeneous cell surface expression while absent on indispensable normal cells. To avoid antigen-negative tumor cell escape, ideal target antigens will have to be associated with cellular properties and functions that are indispensable for tumor progression and metastasis. A candidate CAR target under evaluation in EwS is the ganglioside antigen G_D2_^7^. G_D2_-targeted CAR T cell therapy has resulted in objective responses in patients with neuroblastoma and diffuse midline glioma^8–10^, which express G_D2_ at high levels. In EwS, G_D2_ expression is often low and not consistent among and within individual patients^11^, thus G_D2_-targeted T cells alone are unlikely to fully eradicate the disease. Another surface ganglioside with restricted normal tissue expression is stage-specific embryonic antigen-4 (SSEA-4), a marker of human embryonic stem cells^12^. Whereas SSEA-4 is lost during normal tissue development^13^, it can be aberrantly expressed in cancer, including various carcinomas^14–16^, glioblastoma^17^ and another pediatric bone cancer, osteosarcoma^18^. SSEA-4^high^ expressing cells among bulk tumor cells were found to have a migratory phenotype and higher tumor-initiating potential in in vivo models^18,19^. First attempts were reported to use SSEA-4 as therapeutic immune target for monoclonal antibodies^17,20^ or more recently CAR T cells^16,21^.

To find out whether SSEA-4 has functional relevance in EwS and could thus be a suitable immune target, we studied the functional properties associated with cell surface SSEA-4 in this cancer, along with proof-of-concept experiments demonstrating the sensitivity of EwS cells to SSEA-4-specific CAR T cells.

## Results

### Ganglioside SSEA-4 is expressed in a high proportion of Ewing sarcomas

**Figure 1:**
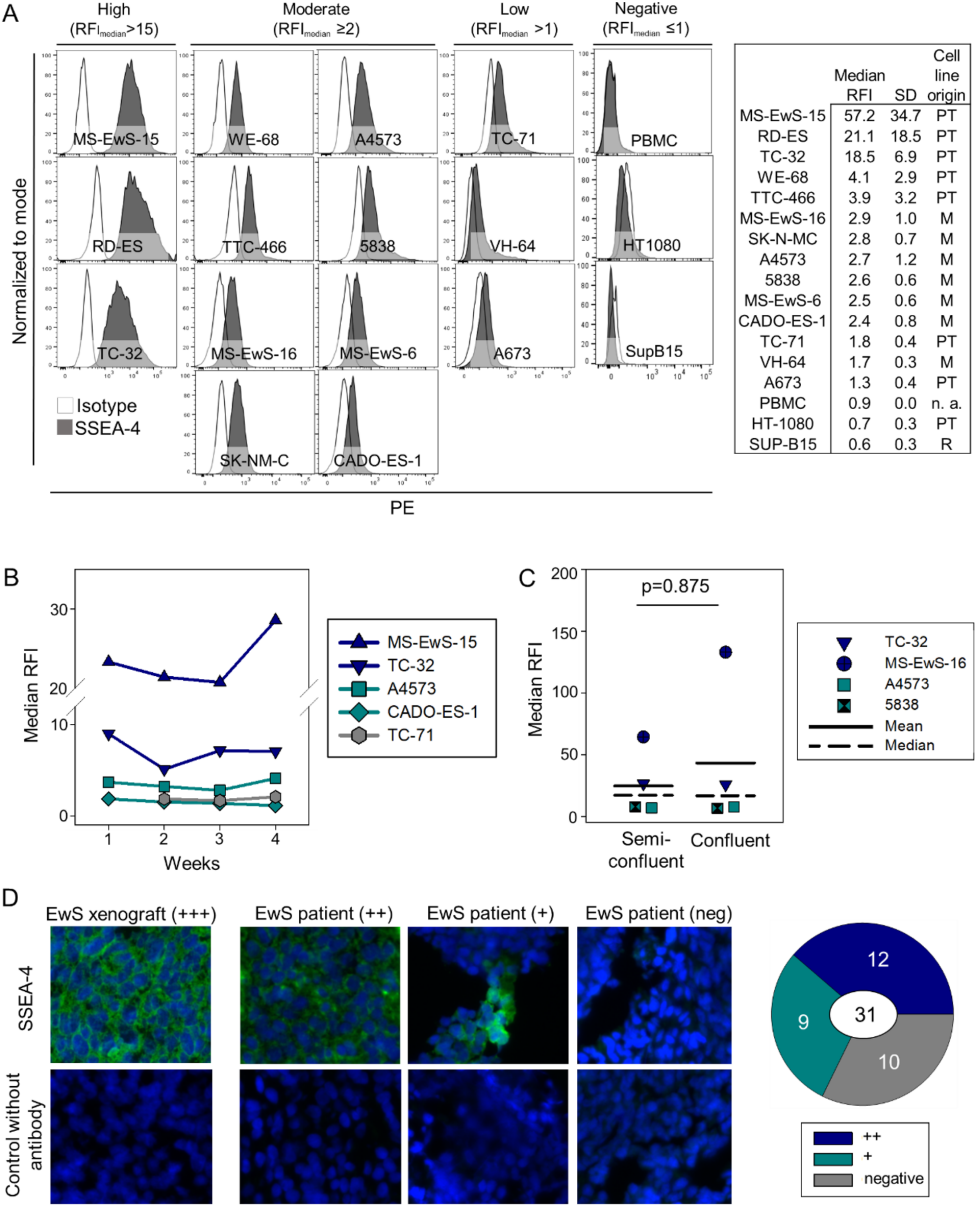
SSEA-4 expression in EwS. (**A**) SSEA-4 surface expression by flow cytometry in 14 EwS cell lines, derived from primary tumors (PT) or metastatic relapse samples (M), as indicated. Median relative fluorescence intensities (RFI) were determined in 2 to 4 individual experiments per cell line and used to calculate the mean and standard deviation (SD) of SSEA-4 expression per cell line as shown in the table. Cell lines were categorized by median RFI as SSEA-4 high (RFI ≥ 15), SSEA-4 moderate (RFI ≥ 2 and < 15) or SSEA-4 low (RFI > 1 and < 2). PBMC from healthy donors and cell lines from a B lineage leukemia (SUP-B15 and a fibrosarcoma (HT-1080) were stained as controls. Representative experiments with PBMC from 2 donors and 4 replicates for the cell lines are shown. (**B**) Stability of SSEA-4 expression during 4 weeks of continuous cell culture of 5 individual cell lines expressing SSEA-4 at high (MS-EwS-15, TC-32), moderate (A4573, Cado-ES-1) or low (TC-71) densities. Differences in SSEA-4 expression over time was analyzed with the one-way repeated measurements ANOVA (p = 0.255). (**C**) SSEA-4 expression in 4 EwS cell lines (MS-EwS-16, TC-32, A4573 and 5838) at cell confluencies of 50–70% (semiconfluent) versus 80–100% (confluent). Differences were analyzed with the Wilcoxon signed rank test. (**D**) Immunofluorescence analysis of SSEA-4 expression in tumor tissues, including a TC-32 murine xenograft tumor and tumor tissues from 31 EwS patients with either primary or relapsed disease, stained with MC 813-70 antibody or without primary antibody as control. Shown are representative examples of tumors with high (++), moderate/heterogeneous (+) and negative SSEA-4 expression. Details regarding the individual patients are found in Supplementary Table 2.

Surface expression of SSEA-4 was determined by flow cytometry in 14 individual EwS cell lines. Using a relative median fluorescence intensity (RFI) > 1 as cut-off to define SSEA-4 positivity, all cell lines expressed SSEA-4 (Fig. 1A). Three cell lines (21.4%) had high densities of surface SSEA-4, defined by an RFI above 15, eight cell lines (57.1%) expressed SSEA-4 at moderate (RFI ≥ 2 and ≤ 15) and three (21.4%) at low densities (RFI > 1 and < 2). No differences in SSEA-4 expression levels were found between cell lines derived from primary tumors versus metastatic relapses (p = 0.165) (Fig. 1A), nor between cell lines with EWSR1-ERG (5838, MS-EwS-16, TTC-466) versus EWSR1-FLI1 fusions (all others) (p = 0.353).

SSEA-4 expression densities during continued in vitro cell culture remained stable for 4 weeks, with only minor fluctuations (Fig. 1B) (p = 0.255). Since surface expression of gangliosides can be substantially affected by cell confluency^22^, we quantified SSEA-4 surface expression in 4 EwS cell lines grown to either semiconfluent (50 to 70%) or confluent monolayers (80 to 100%). In contrast to our findings with an alternative ganglioside, GD2, in osteosarcoma^22^, SSEA-4 expression densities in EwS cells from individual cell lines were comparable regardless of cell confluency (Fig. 1C). Next, we investigated SSEA-4 expression in tumor biopsies, using fluorescence microscopy analysis of cryopreserved tissue sections. To validate immunofluorescence detection of SSEA-4, we used a murine tumor xenograft of the EwS cell line TC-32 which highly expresses SSEA-4 by flow cytometry (Fig. 1A). Immunofluorescence staining using the same anti-SSEA-4 antibody clone as for flow cytometry confirmed high SSEA-4 expression on TC-32 cells also in the xenograft (Fig. 1D). Among tumor tissue sections obtained from 31 EwS patients with either newly diagnosed, pretherapeutic (n = 25) or relapsed (n = 6) disease (Supplementary Table 2), 21 (67.7%) expressed SSEA-4 at high (n = 12) or moderate and/or heterogeneous (n = 9) fluorescence intensities, whereas 10 samples (32.3%) were SSEA-4 negative.

Univariate analysis by Fisher´s exact test did not reveal any significant associations of SSEA-4 expression with clinical disease parameters, including primary versus relapsed disease (p = 0.358), localized versus metastatic disease (p = 0.177), female versus male sex (n = 0.697), tumor localization in trunk versus extremities (p = 0.675) or age up to 14 years versus ≥ 15 years (p = 1.000), nor with survival (p = 0.677), the latter confirmed by Kaplan Meier analysis (Supplementary Fig. 1).

Thus, ganglioside SSEA-4 is expressed in a high proportion of EwS unrelated to the type of EWSR1 gene fusion and to clinical disease parameters and outcome. Surface expression densities on tumor cells remain consistent throughout in vitro propagation under standard cell culture conditions.

### SSEA-4^high^ expression in EwS cells is associated with functional in vitro parameters of aggressive tumor growth

**Figure 2:**
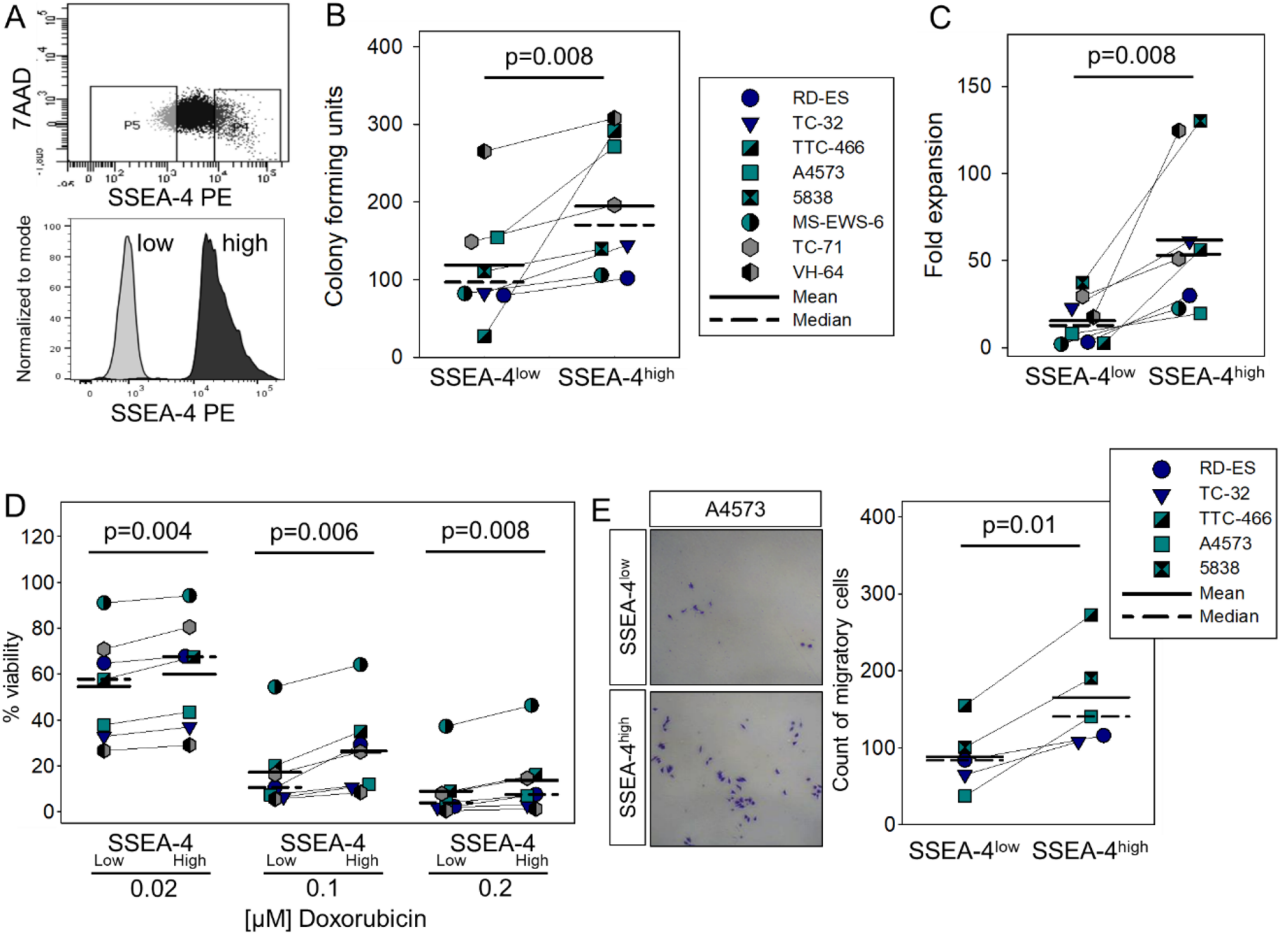
SSEA-4^high^ phenotype in EwS cells is associated with in vitro parameters of more aggressive tumor growth. (**A**) Sorting strategy used to separate SSEA-4^low^ and SSEA-4^high^ subpopulations. EwS cell lines were stained with PE-labeled SSEA-4 specific antibody (clone MC-813–70). 7AAD staining was used to exclude dead cells. Gates were set on 7AADneg and SSEA-4^low^ (10% cells with lowest SSEA-4 expression) and on 7AADneg, SSEA-4^high^ (10% cells with highest SSEA-4 expression) cells. (**B**) Sorted SSEA-4^low^ and SSEA-4^high^ cells, respectively, were seeded at 500 cells/well on ultra-low attachment plates (TC-32) or in methylcellulose containing medium (all others) and the colony forming units (CFU) were assessed after 11 days (TC-32) or 8 days of culture. Shown are the means of 2 independent experiments per cell line. (**C**) Cell viabilities of SSEA-4^low^ and SSEA^high^ sorted cells were compared after 96 h of in vitro culture at 37 °C by quantifying relative luminescence units (RLU) using the Cell Titer-Glo® kit. Normalized RLU were calculated by division of the RLU measured after 96 h by the RLU measured directly after sorting. Shown are the means of 2 individual experiments per cell line. (**D**) Viabilities of SSEA-4^low^ versus SSEA-4^high^ cells following 72-h in vitro culture with the indicated concentrations of doxorubicin. RLU were measured using the Glo-Max® assay. The percentages of viable cells were calculated using the medium control as reference. The experiment was reproduced twice for each cell line, including all cell lines used in (**A**–**C**) except 5838 which was not available in our lab at the time of this experiment. Shown are the means and standard deviations of 7 cell lines. (**E**) Numbers of EwS cells migrating through membrane pores following 24-h starvation in medium without FBS, sorting into SSEA-4^low^ and SSEA^high^ subpopulations and 48-h incubation in transwell chambers with 20% FBS containing medium in the lower chambers. Cells were counted after staining with 0.2% crystal violet. After testing for normal distribution, statistical significances in (**B**) (data not normally distributed) were determined with the Wilcoxon signed rank test and in (**C**–**E**) (normal distribution) with the paired student´s t-test.

Gangliosides are involved in cellular key functions, including proliferation, differentiation, adhesion and cell death, and SSEA-4 was suggested to contribute to tumorigenicity and invasiveness in various cancers^15,17,18^. As a first step to understanding the functional role of SSEA-4 in EwS, we compared in vitro parameters of tumor cell growth in paired subpopulations of tumor cells with low/negative (SSEA-4^low^) versus high (SSEA-4^high^) SSEA-4 expression, selected from 8 EwS cell lines by flow cytometry-based cell sorting (Fig. 2A). Gates were set on 10% cell populations with the highest versus lowest fluorescence intensities of SSEA-4 antibody staining.

To compare colony-forming capacities, freshly sorted SSEA-4^low^ and SSEA-4^high^ tumor cells were cultured in dishes containing semi-liquid methylcellulose medium for 8 days, with the exception of TC-32 cells that fail to form colonies on methylcellulose and instead were cultured on ultra-low attachment plates. Across 8 cell lines, significantly higher numbers of colonies were formed by cells sorted for SSEA-4^high^ compared to SSEA-4^low^ expression (Fig. 2B). A cell viability assay following in vitro cell culture for 4 days revealed a significant proliferative advantage of cells sorted for SSEA-4^high^ compared to SSEA-4^low^ expression (Fig. 2C). Sensitivity to cytotoxic drugs was assessed by viability analysis of sorted EwS cells after 72-h incubation with increasing concentrations of doxorubicin, a drug used in the standard treatment of EwS. Significantly higher proportions of viable, chemoresistant cells were found among EwS cells sorted for SSEA-4^high^ compared to SSEA-4^low^ expression (Fig. 2D). In 5 of the cell lines, we further analyzed the migratory capacities of SSEA-4^low^ and SSEA-4^high^ in transwell-chamber experiments using 20% FBS as attractant. After 48 h, significantly higher numbers of cells from SSEA-4^high^ sorted subpopulations compared to SSEA-4^low^ sorted subpopulations had migrated through the membrane (Fig. 2E).

We conclude that SSEA-4^high^ expression in EwS cells is significantly and consistently associated with functional characteristics of in vitro tumor growth and aggressiveness, including proliferation, colony formation, chemoresistance and propensity to migrate.

### SSEA-4^high^ expression in EwS is not associated with heterogeneous EWSR1-FLI1 expression or epithelial/mesenchymal plasticity

**Figure 3:**
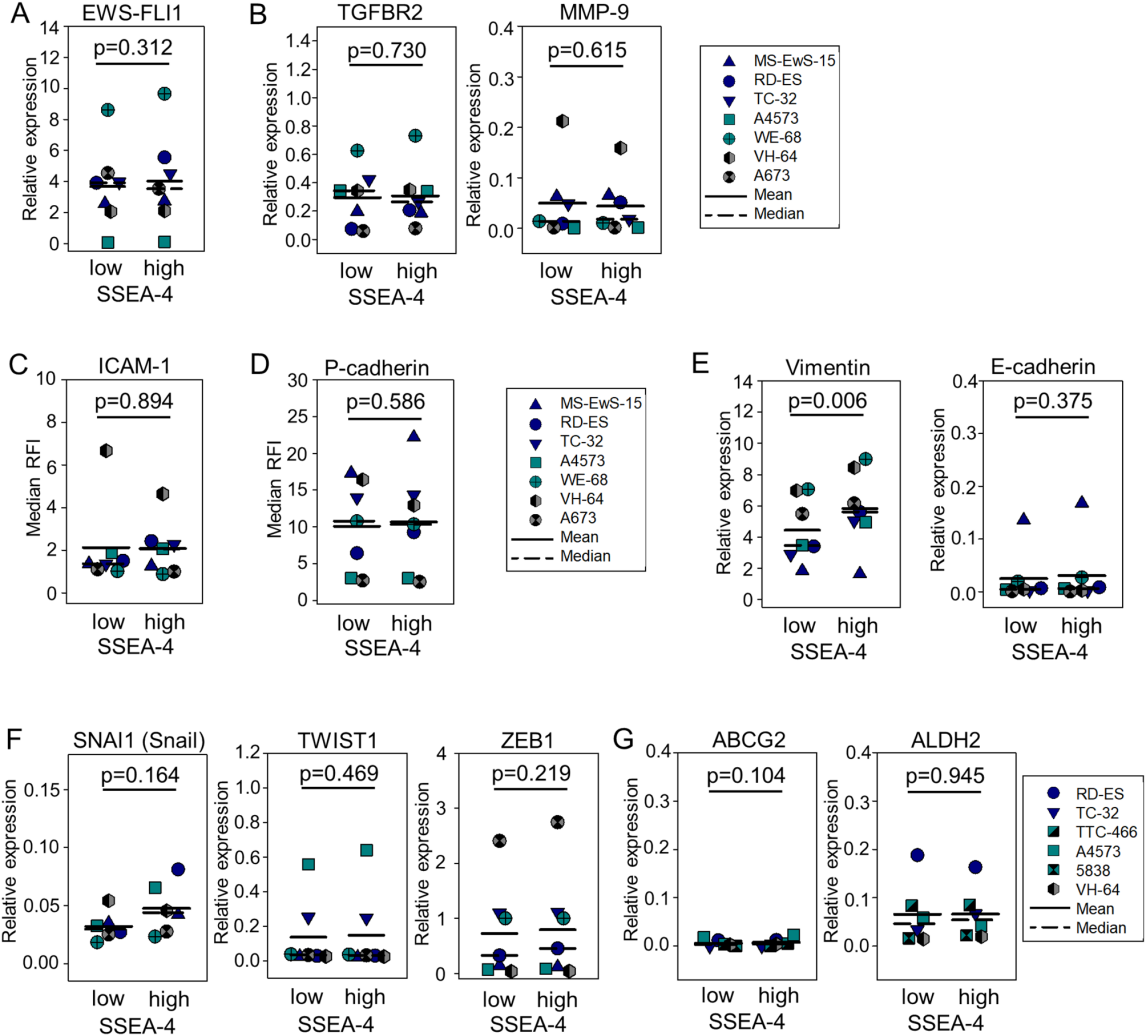
SSEA-4^high^ phenotype is not associated with expression of EWSR-FLI1 and with epithelial/mesenchymal plasticity. (**A**) Expression of the EWSR1-FLI-1 fusion transcript in SSEA-4^low^ and SSEA-4^high^ sorted subpopulations of 7 EwS cell lines was quantified by qRT-PCR. Triplicates of mRNA isolated from 2–3 independent sorting experiments per cell line were analyzed. Relative expression was determined by calculating Delta Ct with a reference gene. (**B**) Gene expression of TGFBR2 and MMP-9 by qRT-PCR analysis as above. (**C**) ICAM-1 and (**D**) P-cadherin surface expression in 7 EwS cell lines by flow cytometry using APC-labeled antibodies against ICAM-1 or anti-P-cadherin, respectively. Costaining with anti-SSEA-4 PE antibody allowed to compare ICAM-1 expression levels between SSEA-4^low^ versus SSEA-4^high^ expressing cells by gating on the 10% lowest and 10% highest SSEA-4 expressing cells. (**E**) Expression of genes encoding Vimentin and E-Cadherin, (**F**) EMT transcriptions factors SNAI1 (Snail), TWIST1 and ZEB1, and (**G**) markers associated with cancer-initiating cells (ABCG2, ALDH2) in SSEA-4^low^ and SSEA-4^high^ subpopulations of 6 or 7 EwS cell lines (TC-32 not analyzed for SNAI1) were quantified by qRT-PCR as described above. After testing for normal distribution, statistical significances for E-cadherin, TWIST1 and ZEB1 (data not normally distributed) were determined with the Wilcoxon signed rank test and in all others (normal distribution) with the paired student’s t-test.

The in vitro characteristics of SSEA-4^high^ cells in EwS, with increased proliferation, drug resistance and motility, are reminiscent of a previously described functional signature of this cancer^23^, determined by fluctuations of expression of the EWSR1-FLI1 fusion transcript, combining both mesenchymal and epithelial features and contributing to metastatic progression and aggressiveness^24^. To investigate a potential association of cell surface SSEA-4 with the genetic driver of the disease, we compared gene expression of the EWSR1-FLI1 fusion transcript and two of its direct targets, transforming growth factor β receptor 2 (TGFBR2) and matrix metalloproteinase 9 (MMP-9), in SSEA-4^low^ versus SSEA-4^high^ EwS cells by qRT-PCR analysis, and surface expression of a third target, intercellular adhesion molecule 1 (ICAM-1), by flow cytometry. Neither EWSR1-FLI1 (Fig. 3A) nor any of the downstream targets (Fig. 3B,C) were differentially expressed between the two subpopulations, arguing against an association of fluctuations in gene expression of the disease-driving oncogene with the SSEA-4^high^ phenotype. We further analyzed differential expression of P-cadherin, a marker and mediator of cancer cell migration and invasion^25^, by flow cytometry. P-cadherin was detected on the cell surface in all 7 EwS cell lines, with comparable expression densities between SSEA-4^low^ and SSEA-4^high^ subpopulations (Fig. 3D). To address a potential relationship between the SSEA-4^high^ phenotype and epithelial/mesenchymal plasticity, we compared epithelial and mesenchymal markers and transcription factors associated with epithelial-to-mesenchymal transition (EMT)-related processes. Whereas the mesenchymal marker vimentin was expressed at significantly higher levels by SSEA-4^high^ than by SSEA-4^low^ subpopulations (p = 0.006) (Fig. 3E), neither E-cadherin (Fig. 3E), an epithelial marker, nor any of 3 EMT-associated transcription factors, Snail, Twist and ZEB1 (Fig. 3F) or markers associated with cancer stem-like cells/cancer-initiating cells (ABCG2, ALDH2) (Fig. 3G) were differentially expressed. Thus, SSEA-4 expression in EwS is not determined by variations of EWSR1-FLI1 expression, nor does it reflect EMT-like plasticity in this cancer.

### SSEA-4 expression increases during in vivo tumor growth, leading SSEA-4^low^ EwS cells to regain SSEA-4^high^ expression in murine tumor xenografts

**Figure 4:**
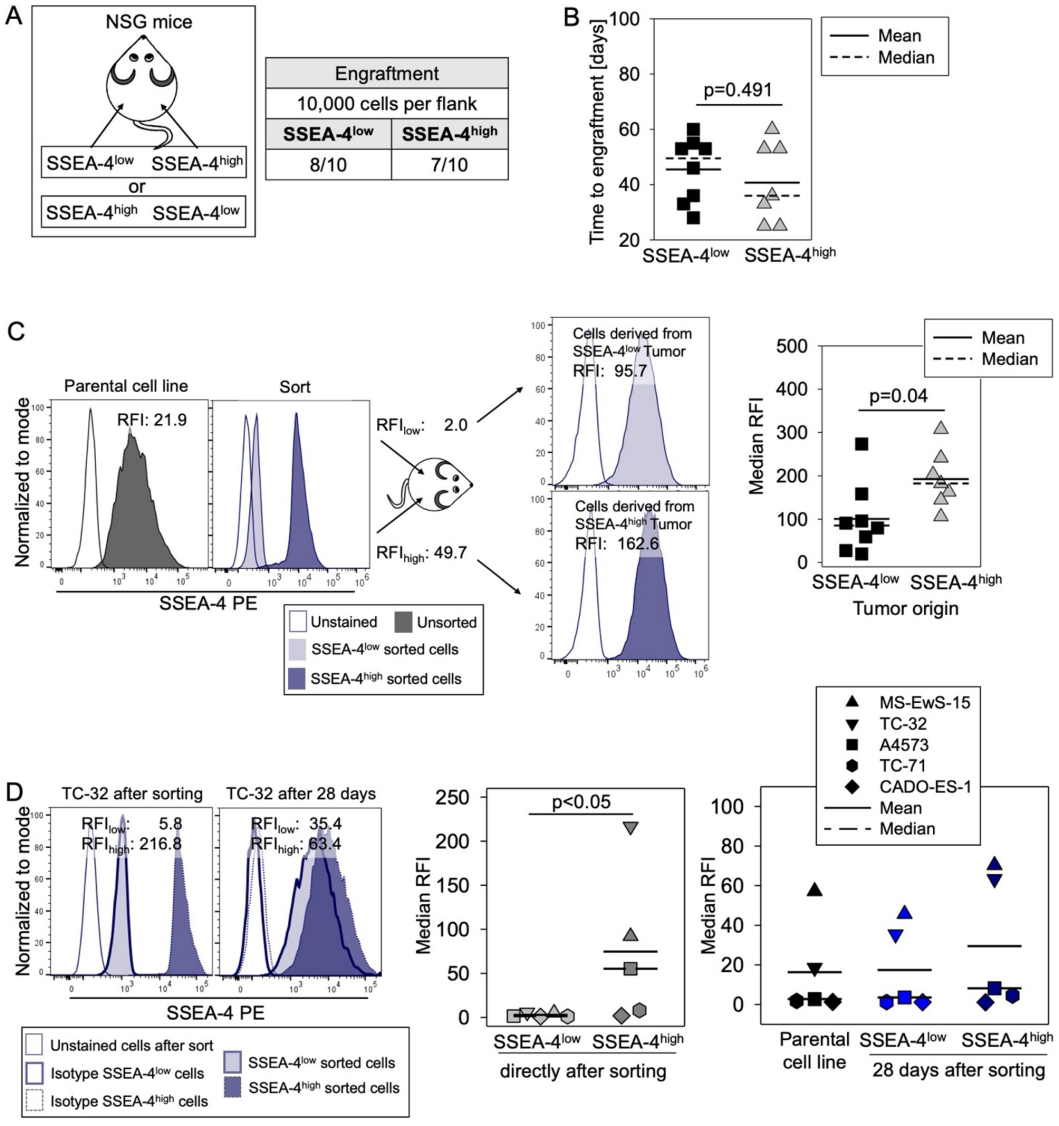
SSEA-4 expression increases during in vivo tumor growth, and SSEA-4^low^ EwS cells regain SSEA-4^high^ expression in vivo and in vitro. (**A**) Engraftment of TC-32 cells in NSG mice after subcutaneous injection of 10,000 SSEA-4^low^ versus 10,000 SSEA-4^high^ TC-32 cells each directly after cell sorting into the left and right flanks of 10 NSG mice, respectively. (**B**) Time to engraftment was assessed via palpation twice per week. The student´s t-test was used to analyze differences. (**C**) SSEA-4 surface expression by flow cytometry on tumor cells from dissociated tumor xenografts derived from either SSEA-4^low^ and SSEA-4^high^ subpopulations. The left panel shows one representative example of SSEA-4^high^ and SSEA-4^low^ cell populations derived from the xenografts compared to SSEA-4 expression after cell sorting directly prior to injection into mice and the parental cell line. The right panel shows the median RFI from all engrafted tumors.The student´s t-test was used to analyze differences. (**D**) SSEA-4 expression levels during 28-day in vitro culture of sorted SSEA-4^low^ and SSEA-4^high^ subpopulations from five EwS cell lines analyzed by flow cytometry. Shown are the SSEA-4 expression quantified by median RFI of the parental cell lines, directly after sorting and on cells from SSEA-4^low^ and SSEA-4^high^ subpopulations after 28 days of continued cell culture. Statistical significance was determined with the Friedman Repeated Measures Analysis of Variance on Ranks (p = 0.003) and subsequent significances were determined with the Tukey Test. Only significant differences are annotated.

To address the functional relevance of SSEA-4 expression for in vivo tumor growth, we compared the capacity of SSEA-4^low^ versus SSEA-4^high^ subpopulations of TC-32 tumor cells, obtained by cell sorting as in Fig. 2A, to initiate tumor xenografts in immunodeficient mice. Of each subpopulation, 10,000 cells each were subcutaneously (s.c.) transplanted into the right versus left flanks of 10 NSG mice (Fig. 4A). Both SSEA- 4^low^ and SSEA-4^high^ TC-32 cells generated tumor xenografts, with 8 (SSEA-4^low^) versus 7 (SSEA-4^high^) tumors arising in 10 mice (Fig. 4A) after a median time to engraftment of 50 days (SSEA-4^low^) versus 36 days (SSEA-4^high^) (p = 0.491) (Fig. 4B). Upon reanalysis of SSEA-4 expression at autopsy, tumors derived from SSEA-4^high^ TC-32 cells had even higher expression levels of SSEA-4 (median RFI 182.2, range 105.9 to 307.6) than the SSEA-4^high^ cell population directly after sort (RFI 49.7). On tumor cells derived from SSEA-4^low^ cells, a considerable increase of SSEA-4 surface expression was observed, which remained significantly lower (median RFI 85.2, range 19.6 to 273.4) than in tumors from SSEA-4^high^ cells (p = 0.04), but reached a median RFI comparable to the SSEA-4^high^ subpopulation directly after sort (Fig. 4C). Thus, SSEA-4 expression increases in both SSEA-4^high^ and SSEA-4^low^ EwS cells during in vivo tumor growth. Tumor cells selected for SSEA-4^low^ expression regain higher SSEA-4 expression during in vivo tumor formation in the murine xenograft model, explaining the observed lack of difference in the biological behaviour of SSEA-4^low^ vs. SSEA-4^high^ subpopulations injected in vivo.

In vitro studies confirm the plasticity of SSEA-4 expression in EwS cells also under in vitro culture conditions and in 4 additional EwS cell lines with variable SSEA-4 expression densities (Fig. 4D). SSEA4^low^ sorted TC-32 cells, the cell line used for the above in vivo experiment, reacquired SSEA-4 expression levels of unsorted TC-32 cells during 28 days of monolayer culture (Fig. 4D, left panel). Sequential analysis of SSEA-4 expression on SSEA-4^low^ cells from 5 EwS cell lines directly after cell sorting (median RFI 1.5, range 0.9 to 5.8) and after 28 days of continuous in vitro cell culture confirmed a regain of SSEA-4 expression (median RFI 3.6, range 1.1 to 45.7) to levels comparable to the unsorted cell lines (median RFI 2.7, range 1.3 to 57.2). In contrast to in vivo growth conditions, SSEA-4 expression on SSEA-4^high^ sorted subpopulations (median RFI 55.2, range 1.9 to 216.8) did not increase during in vitro monolayer growth (median RFI 8.2, range 1.1 to 70.3).

Together, these data demonstrate high stability of SSEA-4 on EwS cells with a regain of higher surface expression levels in SSEA-4^low^ sorted subpopulations both in vitro and in vivo.

### Engineering of T cells with an SSEA-4 specific *CAR* enables effective antigen-specific cytolysis of EwS cells

**Figure 5:**
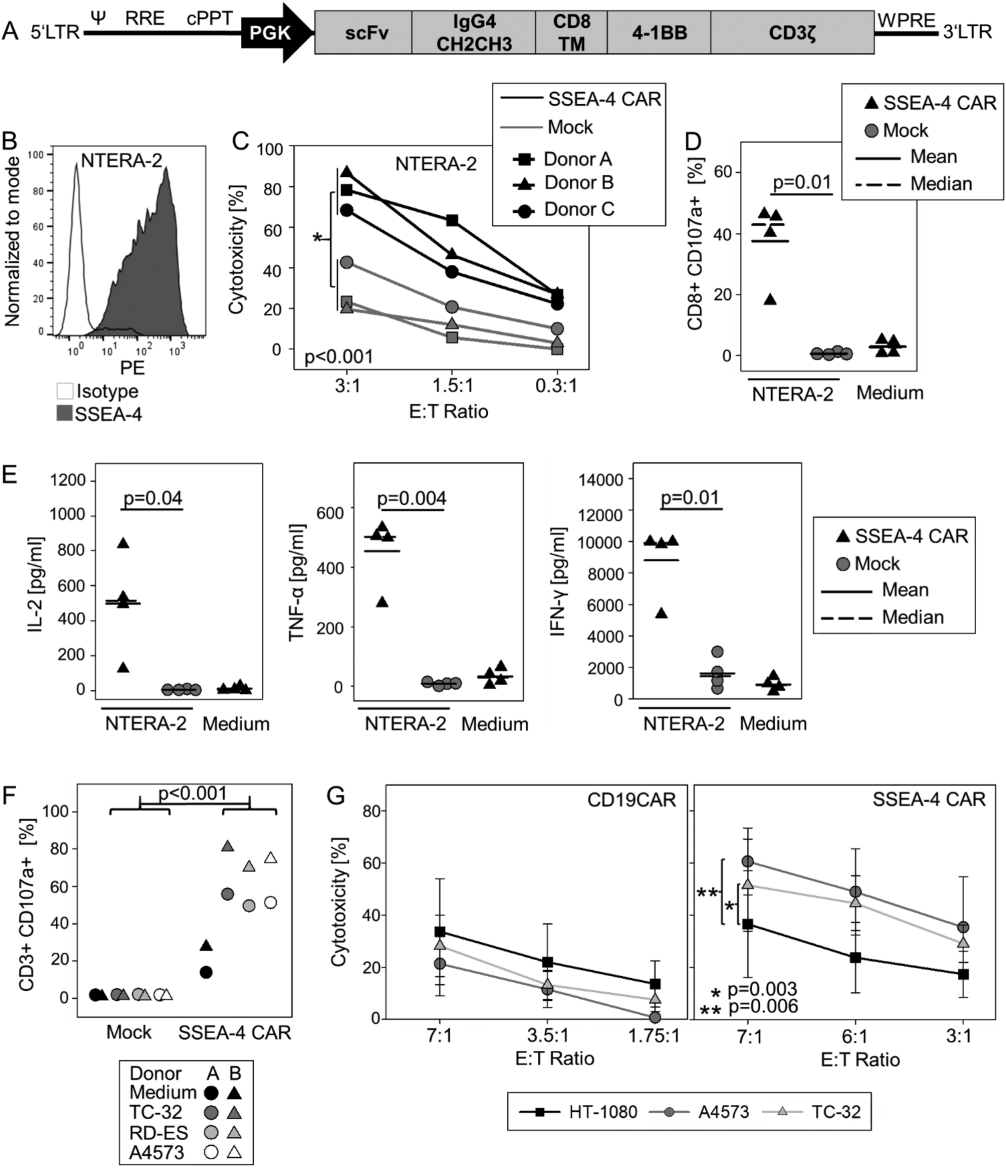
SSEA-4-specific CAR gene-engineered T cells effectively interact with SSEA-4 positive tumor cells in an antigen-specific manner. (**A**) Schematic of second-generation SSEA-4-specific CAR construct. (**B**) SSEA-4 expression of the teratocarcinoma cell line NTERA-2 (median RFI 152.9). (**C**) CAR T-cell mediated target cytolysis. On day 12 of expansion, SSEA-4-specific CAR T cells and non-transduced T cells (mock control) were co-incubated with CellTrace™ Violet-labeled cells from the SSEA-4-positive teratocarcinoma cell line NTERA-2 at the indicated E:T-ratios. After 24 h, the fraction of viable NTERA-2 cells was quantified by flow cytometry. Shown are results from 3 donors and the means of triplicates. (**D**) Degranulation response of SSEA-4-redirected CAR T and non-transduced control T cells (mock) to coincubation with NTERA-2 cells at a ratio of 1:1 by CD107a expression quantified by flow cytometry. CAR T cell cultures without target cells (medium) were used as controls. Analysis was performed on the CD3 + population for non-transduced control T cells and on the CAR + CD3 + subset for CAR-transduced T cells. Shown are results for 4 donors and the means of duplicates. (**E**) Cytokine release by CAR T cells cocultured with NTERA-2 cells on day 11 following transduction at a ratio of 1:2 for 24 h by analysis of supernatants using the MACSPlex technique. Cultures of non-transduced T cells with tumor cells and CAR T cells without stimulation served as controls. Shown are results from 4 donors and the means of duplicates. (**F**) Degranulation response of SSEA-4-redirected CAR T and non-transduced control T cells (mock) to coincubation with the indicated EwS cell lines by CD107a expression quantified by flow cytometry. Analysis was performed on the CD3 + population for non-transduced control T cells and on the CAR + CD3 + subset for CAR-transduced T cells. Shown are results for 2 donors with the means of triplicates. (**G**) Cytolysis of SSEA-4 positive EwS cell lines (A4573, TC-32) versus SSEA-4 negative target cells (HT-1080) by SSEA-4 CAR-transduced T cells or control T cells transduced with an analogous CD19-specific CAR determined in a calcein release assay. Shown are the means and standard deviations of biological replicates from 3 donors. Significances were analyzed using a paired student’s t-test over all E:T ratios (**C**, **G**). Only significant differences are annotated.

Due to its high surface expression in a substantial proportion of EwS, association with features of cancer aggressiveness and chemoresistance and stability of expression, along with the reported restrictive expression in normal human tissues^13^, SSEA-4 could be an attractive immunotherapeutic target. To retarget human T cells against SSEA-4, we designed a second-generation CAR with epitope specificity for the binding domains of the anti-SSEA-4 monoclonal antibody clone REA-101 (Fig. 5A). Lentiviral transduction of in vitro activated human T cells from 4 donors resulted in reliable expression of the CAR in 34.7 to 55.1% (median 44.0%) of CD3 + T cells. In proof-of-concept experiments, SSEA-4 specific CAR T cells specifically interacted with cells from the SSEA-4 positive teratocarcinoma cell line NTERA-2 (Fig. 5B), resulting in efficient cytolysis of tumor cells (Fig. 5C), target-induced degranulation responses by CD107a upregulation (Fig. 5D) and secretion of IL-2, TNF-α and IFN-γ in a strictly antigen-specific manner (Fig. 5E). Next we assessed the capacity of SSEA-4-specific CAR T cells to target EwS cell lines with variable SSEA-4 expression. Coculture of T cells from two additional donors, transduced with the SSEA-4 specific CAR at efficiencies of 27.0% and 28.9%, respectively, with SSEA-4-positive EwS cells resulted in antigen-dependent degranulation responses (Fig. 5F) and cytolysis of SSEA-4-expressing EwS cells (Fig. 5G). Control CAR T cells directed against CD19, which is not expressed in EwS, exerted only low background cytolytic responses against SSEA-4-positive targets, and background cytolysis of an SSEA-4 negative target cell line was limited and comparable with SSEA-4- and CD19-specific CAR T cells.

Overall we conclude that CAR targeting of SSEA-4 could be an attractive strategy to eliminate tumor cell subsets with high propensity to drive disease progression in EwS and therefore deserves further evaluation towards clinical translation.

## Discussion

Immune therapy of cancer with CAR-engineered T cells has emerged as an attractive novel treatment modality but is limited in many solid cancers by a scarcity of adequate target antigens. In this report, we provide evidence that the cell surface ganglioside SSEA-4 has a significant biological role in EwS and thus could serve as a valuable therapeutic target in this cancer. First, we found SSEA-4 to be expressed in a high proportion of these cancers. At least moderate levels of SSEA-4 were detected in about two thirds of cell lines and primary tissues, including samples from relapsed disease, and low surface expression clearly above background was identified in all remaining cell lines. Second, SSEA-4^high^ expression was associated with functional attributes of a particularly aggressive behaviour, including the capacity to proliferate at higher rates, higher clonogenicity and higher migratory capacities, along with increased chemoresistance against a key agent used in standard EwS therapy. Third, SSEA-4 expression on EwS cells was highly robust during continued cell culture and upon in vivo xenografting, with increasing expression levels during in vivo growth of xenografts and spontaneous regain of SSEA-4^high^ expression on cell subsets with low expression selected from bulk cultures.

An association of SSEA-4 expression with tumor growth and invasiveness is supported by published evidence also in other cancers^15,17,18^. Posttherapeutic tumor tissues in patients with osteosarcoma had higher proportions of SSEA-4^high^ tumor cells than initial biopsies, suggesting higher chemoresistance of SSEA-4^high^ cells^18^. Similar findings were made in patient-derived breast cancer xenografts where high expression of SSEA-4 was found in tumors derived from residual cells after neoadjuvant chemotherapy and from relapse samples^15^. In prostate cancer, SSEA-4^high^ cell subpopulations were reported to be highly tumorigenic and characterized by increased cellular adhesion and a migratory phenotype, suggesting a role in cancer invasiona dn metastasis^19^. Spontaneous reacquisition of SSEA-4 has confounded experiments aiming to assess differences in tumor initiation by SSEA-4^low^ and SSEA-4^high^ tumor cells in this work and by others. Stable regain of SSEA-4 expression under in vivo xenograft conditions was first reported in an SSEA-4 negative teratocarcinoma cell line^26^, then dynamic plasticity of SSEA-4 expression with resynthesis in vitro and after in vivo xenografting was reported in many additional solid cancer cell lines, including breast, ovarian and prostate cancer^19^.

Despite the consistent association of SSEA-4^high^ expression status with all investigated parameters of aggressive tumor growth and invasive behaviour, we did not identify any correlations with clinical parameters of disease nor a link to outcome in our patient cohort. This is in contrast to data obtained in patients with osteosarcoma^18^ or breast or ovarian cancer^15^ where SSEA-4 emerged as biomarker of unfavorable disease in clinical cohorts, at least in retrospective studies. One explanation is the high heterogeneity of our retrospective patient population, representing various disease manifestations and consequently small numbers for each subpopulation. Large prospective studies will be needed to answer the question whether SSEA-4 expression status is a predictive biomarker of unfavorable clinical outcome in this cancer.

Recent evidence in EwS supports a disease model in which this mesenchymal cancer can undergo phenotypic and functional changes reminiscent of EMT, to drive disease progression and carcinoma-like metastatic behavior^24^. SSEA-4^high^ EwS cells functionally resemble an intermediate cell type within this model which combines features of proliferation along with increased self-renewal, drug resistance, invasion and motility^23^. Findings in epithelial cancers also support an association between SSEA-4 and EMT^15,19^. Experimental induction of EMT in breast cancer resulted in expression of SSEA-4^15^, and SSEA-4-positive subpopulations in prostate cancer were characterized by a gain of mesenchymal markers and expression of EMT-associated transcription factors along with migratory structures and strong tumorigenic ability^19^. Despite the similarity of the functional phenotype of SSEA-4^high^ cells in EwS with the described intermediate cell type, our data argue against SSEA-4 as a marker of this population. First, SSEA-4 surface expression is not associated with (low) expression of EWSR1-FLI1 or its downstream targets, previously shown to determine epithelial/mesenchymal plasticity in this cancer^24^. Moreover, with the exception of the mesenchymal marker vimentin, we did not find any associations of SSEA-4^high^ expression with markers of EMT and thus no consistent evidence supporting a role of this ganglioside in epithelial/mesenchymal plasticity in EwS.

Overall, the consistent association of SSEA-4^high^ expression with particularly aggressive cellular behavior and its stability and reemergence in xenografts from negatively-selected cells strongly support the use of SSEA-4 as therapeutic target in EwS. Our proof-of-concept experiments demonstrate that an SSEA-4 redirected CAR allows efficient recognition and lysis of SSEA-4-expressing EwS cells. In one previous publication, an alternative SSEA-4 directed CAR was effective to retarget human T cells to pancreatic cancer xenografts in a murine model^16^. One concern remains potential on-target/off-tumor toxicity in human patients. In two recent reports, including one with the same CAR as we used in this work, administration of SSEA-4-specific CAR T cells in in vivo models resulted in significant disease symptoms and death^21,27^. While histologic analyses failed to identify any specific organ damage, SSEA-4 was detected on subpopulations of hematopoetic precursor cells in bone marrow and on vascular progenitor cells and mesenchymal stem cells in the lungs, along with evidence for CAR T cell activation at these two organ sites. Thus, on-target interaction with SSEA-4 mesenchymal stroma cell subpopulations which can be found in almost all adult tissues^28^ are likely responsible for the in vivo toxicity in mice and could cause substantial adverse events in humans. A potential solution is the use of smarter CAR technologies, enabling T cell activation only by coengagement of two independent antigens aberrantly coexpressed on the malignant cells but not in healthy tissues^29,30^.

In summary, we report that SSEA-4, an embryonal ganglioside with restricted tissue expression, is expressed in a high proportion of Ewing sarcomas. SSEA-4^high^ expression in this cancer is associated with functional characteristics of higher aggressiveness and is stable during continuous in vitro culture and during in vivo tumor growth. Proof-of concept experiments confirm effective cytolysis of Ewing sarcoma cells by SSEA-4 redirected CAR T cells. While logic-gated approaches may be required to avoid on-target toxicities of CAR T cells against embryonal markers, SSEA-4 is an attractive target to be further studied as valuable immune target for future advanced T cell therapeutics against EwS and potentially other cancers.

## Methods

### Cell culture

EwS cell lines were grown as adherent cultures on collagen-coated flasks in RPMI 1640 medium supplemented with 10% heat-inactivated FBS (Thermo Fisher, Dreieich, Germany) and 2 mM L-glutamin (Sigma-Aldrich, Taufkirchen, Germany) and maintained at 37 °C and 5% CO_2_. TC-32, TTC-466, 5838, A4573 were gifts from the Children’s Hospital Los Angeles, United States. CADO-ES-1 (#ACC 255), RD-ES (#ACC 260), TC-71 (#ACC 516) and NTERA-2 (#ACC 527) were purchased from DSMZ (Braunschweig, Germany). A673 (#CRL-1598) and SK-N-MC (#HTB-10) from ATCC (Manassas, Virginia, USA). Cell lines MS-EwS-6, MS-EwS-15 and MS-EwS-16 were established in our institution, as reported previously^31^, and WE-68 and VH-64 were gifts from the Institute of Experimental Orthopedics of our institution. Control cell lines SUP-B15 (B lineage leukemia) (#ACC 389) and HT-1080 (fibrosarcoma) (#ACC 315) were purchased from DSMZ and were cultivated in RMPI 1640 medium supplemented with 10% heat-inactivated FBS and 2 mM l-glutamin. Short tandem repeat (STR) profiling was used to confirm the identity of all cell lines (Supplementary Table 1), and all cell lines were regularly checked by PCR to exclude mycobacterial contamination.

### Flow cytometry

For analysis of cell surface expression of SSEA-4, 200,000 or 500,000 cells were stained with PE-labeled anti SSEA-4 antibody (clone MC-813-70, #330406, Biolegend, Heidelberg, Germany or clone REA101 #130-122-958, Miltenyi, Bergisch Gladbach, Germany) or anti-IgG3–PE (clone MG3-35, #401320, Biolegend or clone REA293, #130-113-438, Miltenyi) as isotype control. P-cadherin was analyzed using an APC-conjugated antibody (clone 106020, #FAB761A, R&D, Minneapolis, USA) versus an IgG2a isotype control (clone 20102, #IC003A, R&D), and intercellular adhesion molecule 1 (ICAM-1) with an APC-labeled antibody (clone HA58, #353112) and respective IgG1 control (clone MOPC-21, #400120, both from Biolegend). CAR T cells were stained with fluorescence-conjugated antibodies against CD3 (clone SK7, #344806), CD4 (clone SK3, #344622, both Biolegend) and CD8 (clone RPA-T8, #560774, BD Pharmingen, Heidelberg, Germany) and FITC-labeled goat-anti-human Fcγ Ab (#109-095-098, Jackson ImmunoResearch, Pennsylvania, USA). Samples were acquired directly or within 24 h after staining and fixation with paraformaldehyde. At least 5,000 cells per sample were analyzed with FACS Canto or Diva 9.0 software using FACS Celesta flow cytometers (BD Biosciences, Germany). FlowJo v10 (FlowJo LLC, USA) software was used for data analysis. To calculate relative median fluorescence intensities (RFI), the median fluorescence intensities of stained cells were divided by median fluorescence intensities obtained with isotype antibodies or unstained controls.

### Cell sorting

For FACS sorting, 2 × 10^7^ cells were harvested and stained with anti-SSEA-4-PE as above. Dead cells were excluded by staining with 7AAD (#420404, Biolegend). Unstained control cells (500,000 cells) were used to identify the tumor cell population using side scatter analysis. Gates were set on viable 7AAD-negative cells with the 10% lowest (SSEA^low^) and the 10% highest (SSEA^high^) SSEA-4 expression. Sorted cells were directly analyzed or cultured for up to 28 days in media now containing 1% Penicillin-Strepavidin (Sigma Aldrich).

### Patient material and immunohistochemistry

Cryopreserved tumor biopsies were available from 31 EwS patients (Supplementary Table 2). All patients were included into the multicenter clinical trials E.U.R.O Ewing 99 or EWING 2008 (EudraCT 2008-003658-13), approved by the Institutional Review Board (Az. 2008-391-f-A). Patients and/or their legal guardians had consented to the use of biopsy material for research purposes in accordance with the Declaration of Helsinki.

Slides were dried and fixed in 1% paraformaldehyde (PFA) for 2 min at 4 °C, then washed and permeabilized for 15 min in PBS with 1% BSA and 0.075% Tween. After 3 rounds of washing, slides were rinsed with PBS containing 1% BSA, then incubated for 16 h with primary unconjugated antibody SSEA-4 (clone MC-813-70, #330402, Biolegend), diluted at 1:50 in PBS with 0.01% Tween. As control, a second tissue slide from the same patient was processed without the primary antibody. After 5 washiung steps in TBS-T solution, anti-mouse HRP-polymer (#ARH1001EA, Akoya Bioscience, USA) was added for 10 min at RT. After washing, tyramide signaling amplification (TSA) staining reagent Opal 520 (#FP1487001KT, Akoya Bioscience) was added at 1:100 and slides were incubated for 10 min in the dark at RT, then washed again. Cell nuclei were stained with the fluorescence dye 4ʹ,6-Diamidin-2-phenylindol (DAPI, #GTX16206, GeneTex, USA) for 1 min. Finally, slides were washed and covered with Prolong Diamond Antifade (#36970, Invitrogen, Germany). Imaging was performed with Vectra® 3.0 system (Perkin Elmer, Rodgau, Germany) and InForm Analysis Software (InForm 2.1.4, Akoya Bioscience). Samples were independently evaluated by two investigators, S.K. and W.H.

### Colony formation assay

Tumor cells directly after cell sorting were plated in triplicates at 500 cells/well in 35-mm tissue culture dishes (Thermo Scientific, Waltham, USA) in methylcellulose-enriched media (1.9% methylcellulose, 15% fetal bovine serum, 0.23% BSA, 1% penicillin/streptomycin and 82% Iscove’s modified Dulbecco’s media), then incubated for 7–8 days at 37 °C and 5% CO_2_. TC-32 cells were cultured in ultra-low attachment plates (#3471, Corning, Kaiserslautern, Germany) for 11 days. Numbers of colonies from each culture dish were calculated using an inverted microscope and a scoring grid (Carl-Zeiss, Jena, Germany). The mean colony numbers (colony-forming units, CFU) of triplicate dishes were used for analysis.

### Proliferation assay

Short-term proliferation was analyzed using the CellTiter Glo® Luminescent Cell Viability Assay (#G7570, Promega, Walldorf, Germany). Tumor cells directly after cell sorting were seeded in triplicates at 500 cells per well in two collagen-coated white bottom 96-well plates. One plate was incubated for 30 min at RT to measure the relative luminescence units (RLU) on day 1, the other for 4 days at 37 °C, 5% CO_2_. Cell Titer Glo® reagent was added and the amount of metabolized ATP quantified by determining the RLU with GloMax Discover (Promega). Normalized RLU was determined by dividing the mean RLU of 6 wells (RLU_Mean_) of the day 5 plate by the RLU_Mean_ of the day 1 plate.

### Migration assay

Tumor cells were starved for 18 to 24 h in RPMI medium without FBS and l-glutamin prior to sorting. Transwell plates with 8 µm membrane pores (#3428, Corning) were prepared by coating the lower bottom chamber wells with rat collagen (#Z-17C03-C, Cell Concepts, Umkirch, Germany). RPMI supplemented by 20% FBS and l-glutamin (750 µl/well) was added as attractant. Tumor cells were added at 50,000 cells/well, with two technical replicates per experiment. Migration into the lower chamber was assessed after 48 h of incubation at 37 °C, 5% CO_2_. Migrated cells that adhered to the bottom of the well were fixed with 70% ethanol and incubated for 5 min, then visualized by staining with 0.2% crystal violet for 5 min followed by cell counting under an inverted microscope using a grid.

### Chemosensitivity assay

Tumor cells (5000 cells/well) were seeded into uncoated (A4573) or collagen-coated (all others) opaque-walled 96-well plates. After 24 h, increasing concentrations of doxorubicin or medium as control were added in triplicates. After 72 h, Cell Titer Glo® reagent was added to each well and RLU were quantified as described above. Percent viability was determined by dividing the RLU of doxorubicin-treated cells with untreated cells × 100.

### In vivo experiments

Mouse experiments were approved by the animal care committee of the local government (LANUV, Recklinghausen, Az. 81-02.04.2020.A296). NSG mice were purchased from Charles River (Cologne, Germany) and used for own breeding in the central animal experimental facility Muenster. Animals were housed in pathogen-free rooms in type-2L individually ventilated cages (Charles River) with a maximum of 6 mice per cage, with access to sterile food and water ad libitum and a constant RT at 21 °C. NSG mice of both genders and 7–12 weeks old were used for the experiments. To establish tumor xenografts, 10,000 TC-32 cells directly after cell sorting were injected s.c. into the flanks of 10 animals. Time to engraftment was monitored via palpation twice per week and tumor growth was measured with a caliper. When the tumor volumes reached the experimental endpoint, mice were anesthetized with isoflurane and sacrificed. If no tumor was detectable, mice were sacrificed after 12 weeks. Tumors were immediately dissociated in PBS and singularized by passage through a cell strainer, followed by incubation with red blood cell lysis buffer (Qiagen, Hilden, Germany) and flow cytometry.

### Quantitative real-time PCR (qPCR)

The RNeasy-Kit (Qiagen) was used to isolate RNA, followed by assessment of RNA concentration and purity using Nanodrop analysis (Thermo Scientific). The purity of all samples was above 1.8 A_260_/A_280_. For cDNA synthesis, the Quick-Start Protocol of QuantiTect Reverse Transcription Kit (Qiagen) was applied. For PCR reactions, 1 µl cDNA, 9 µl Luna Universal qPCR Mix (New England Biolabs, Frankfurt am Main, Germany) and 0.5 µM primers were combined. Primers for the reference gene HPRT1 (forward primer 5ʹ-TGAGGATTTGGAAAGGGTGT, reverse primer 5ʹ-GAGCACACAGAGGGCTACAA) and for EWSR1-FLI1 (forward primer 5ʹ-CAGCCTCCCACTAGTTACCC, reverse primer 5ʹ-GGTGAGGCCAGAATTCATG) were purchased from Invitrogen, Carlsbad, CA. For transforming growth factor β receptor 2 (TGFBR2) (Hs_TFGBR2_1_SG), matrix metalloproteinase 9 (MMP9) (Hs_MMP9_1_SG), TWIST1 (Hs_TWIST1_1_SG), SNAI1 (Hs_SNAI1_1_SG), ZEB1 (Hs_ZEB1_2_SG) vimentin (Hs_VIM1_1_SG), E-Cadherin (Hs_CDH1_1_SG), ABCG2 (Hs_ABCG2_1_SG) and ALDH2 (Hs_ALDH2_1_SG) QuantiTect Primer Assays from Qiagen were used. Amplification was performed in triplicates at 95 °C for 1 min, then 95 °C for 15 s and 42 cycles of 60 °C (30 s) and 65 °C (30 s) on a CFX96 Thermal Cycler (BioRad, Feldkirchen, Germany), and analyzed using the SYBR mode. To ensure equal amplification efficiencies, cDNA concentrations were adjusted to threshold cycle values of the HPRT1 reference gene, which were determined using CFX Manager (BioRad). The triplicates of each run were averaged for analysis. Relative gene expression levels were calculated by using the Delta Ct with the formula 2^Ct(HPRT01) − Ct(gene of interest)^.

### *CAR* construct and transduction of human T cells

An SSEA4-specific CAR was assembled by cloning the single-chain antibody domain (scFv) of the monoclonal antibody REA101^21^, with a (G_4_S)_3_ linker between the VL and the VH domains, upstream of a human IgG4 CH2-CH3 hinge/spacer domain (Uniprot P01861; Aa 98-326) followed by the transmembrane domain of human CD8α (Uniprot P01732; Aa 183-206), the intracellular 4-1BB costimulatory domain (Uniprot Q07011; Aa 214-255) and the CD3ζ signaling domain (Uniprot P20963; Aa 52-164). The CAR gene was subcloned into the AgeI and XhoI sites of the retroviral vector pSFG or into the lentiviral vector pMDG2. The CD19-specific CAR used to generate control CAR T cells against an antigen not expressed on EwS cells was previously described^32^. Production of recombinant virus and activation, transduction and expansion of T cells was performed as in previous work^33,34^.

### CD107a degranulation assay

T cells on days 13 or 14 of in vitro culture were incubated with target cells at a stimulator-to-target cell ratio of 1:1 in 200 µl volumes in Eppendorf tubes for 3 h at 37 °C and 5% CO_2_ in the presence of Monensin (#420701, Biolegend) at 1 µl/ml and CD107a-PE antibody (clone H4A3, #328608, Biolegend), then analyzed by flow cytometry.

### Cytotoxicity assay

Tumor cytolysis was assessed by labeling of tumor cells with 2.5 µM CellTrace™ Violet (#C34571, Thermo Fisher) and analysis of viable cells after coincubation with CAR T cells by flow cytometry or with the calcein-AM release assay. In detail, target cells were incubated with calcein-AM at 10 μM (#65-0853-39, Thermo Fisher) for 30 min at 37 °C, washed thoroughly, then coincubated in triplicates in flat bottom 96-well microtiter plates (#CLS3997-50EA Corning, Germany) with CAR T cells at effector-to-target (E:T) cell ratios from 40:1 to 10:1. Additional triplicate wells were set up to assess spontaneous (target cells alone in complete medium) and maximum release (target cells in medium plus 9% Triton X-100). After 4 h at 37 °C in 5% CO_2_, samples were transferred to black-walled 96-well microtiter plates (#165305 Thermo Fisher) and analyzed using the GloMax® Discover multi-mode microplate reader (Promega) at excitation 475 nm and emission 500–550 nm. Data were expressed as arbitrary fluorescent units (AFU). Specific lysis was calculated by using the formula [(test release − spontaneous release)/(maximum release − spontaneous release)] × 100.

### Cytokine analysis

Cytokine secretion by CAR transduced T cells following antigen stimulation was assessed on day 11 following transduction by coculture with NTERA-2 cells at a ratio of 1:2 for 24 h. Culture supernatants were analyzed for cytokine release using the MACSPlex human Cytokine 12 Kit (#130-099-169, Miltenyi) assay according to the manufacturer’s recommendations.

### Statistical analysis

Data were analyzed and visualized using Systat SigmaPlot 11.0 software (Systat Software, San Jose, CA). Continuous variables are described by mean and standard deviation. Categorical variables are described by absolute and relative frequencies. Statistical testing was performed using parametric and non-parametric methods. Details are given in figure captions, respectively. Results were considered to be statistically significant at p ≤ 0.05. The Kaplan–Meier method^35^ was used to analyze overall survival rates with the Log Rank Test.

### Ethics declaration

All methods were carried out in accordance with relevant guidelines and regulations. All methods are reported in accordance with ARRIVE guidelines (https://arriveguidelines.org).

### Supplementary Information

Supplementary Figure 1.

Supplementary Figure 2.

Supplementary Table 1.

Supplementary Table 2.

## Data Availability

Additional data beyond the data found in the article are available from the corresponding author on reasonable request.
