# Supplementary figures and images for: Ganglioside SSEA-4 in Ewing sarcoma marks a tumor cell population with aggressive features and is a potential cell-surface immune target
Source: Sci Rep. 2024 May 24;14:11935. doi: 10.1038/s41598-024-62849-8 (PMC11126692; doi:10.1038/s41598-024-62849-8)

## Slide 1
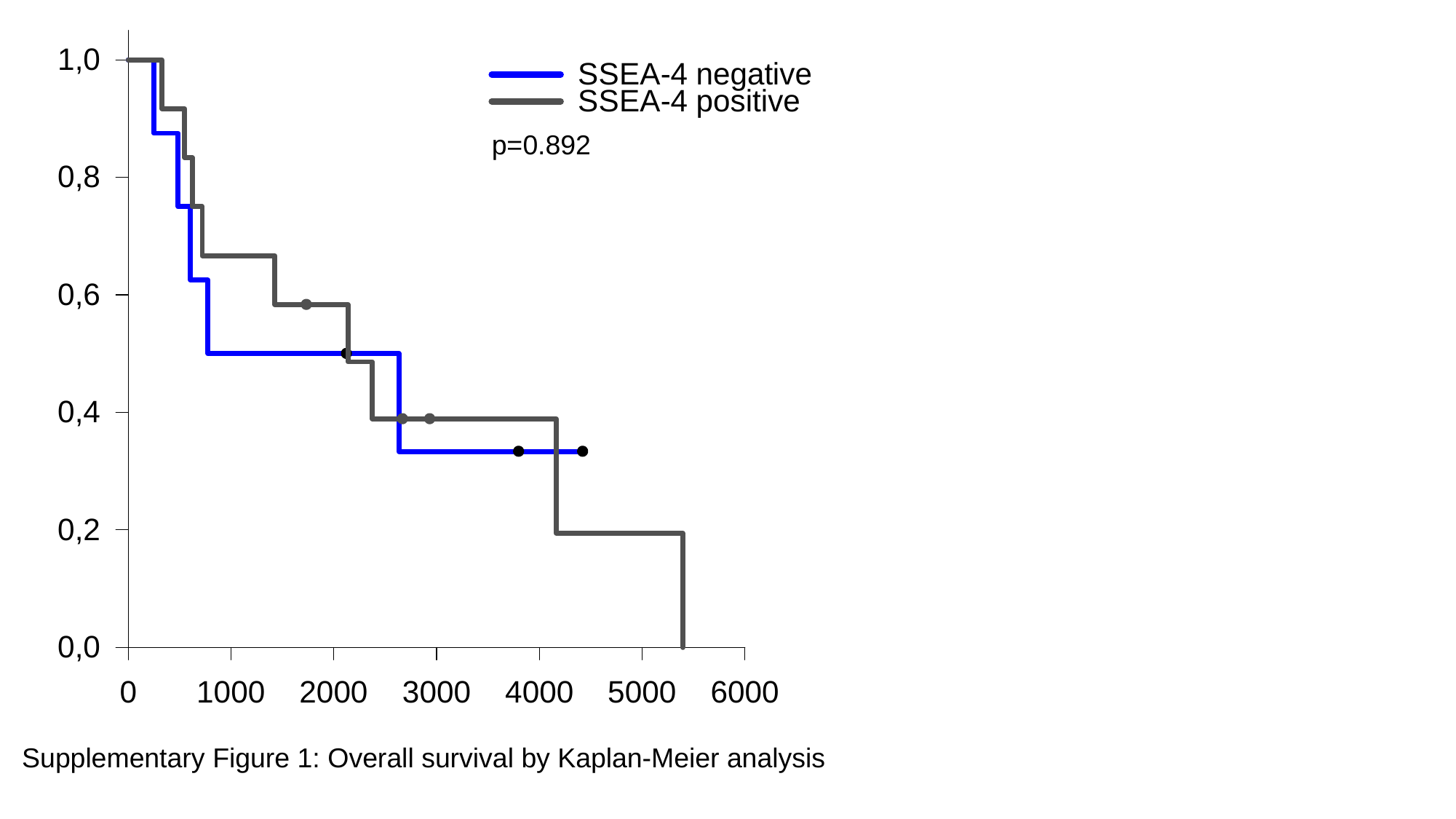

p=0.892
Supplementary Figure 1: Overall survival by Kaplan-Meier analysis

Supplement: Supplementary file 1 — Supplementary Figure 1. [file 41598_2024_62849_MOESM1_ESM.pptx]
