# Supplementary material for: Ganglioside SSEA-4 in Ewing sarcoma marks a tumor cell population with aggressive features and is a potential cell-surface immune target
Source: Sci Rep. 2024 May 24;14:11935. doi: 10.1038/s41598-024-62849-8 (PMC11126692; doi:10.1038/s41598-024-62849-8)
Supplement: Supplementary file 2 — Supplementary Figure 2. [file 41598_2024_62849_MOESM2_ESM.pptx]

## Slide 1
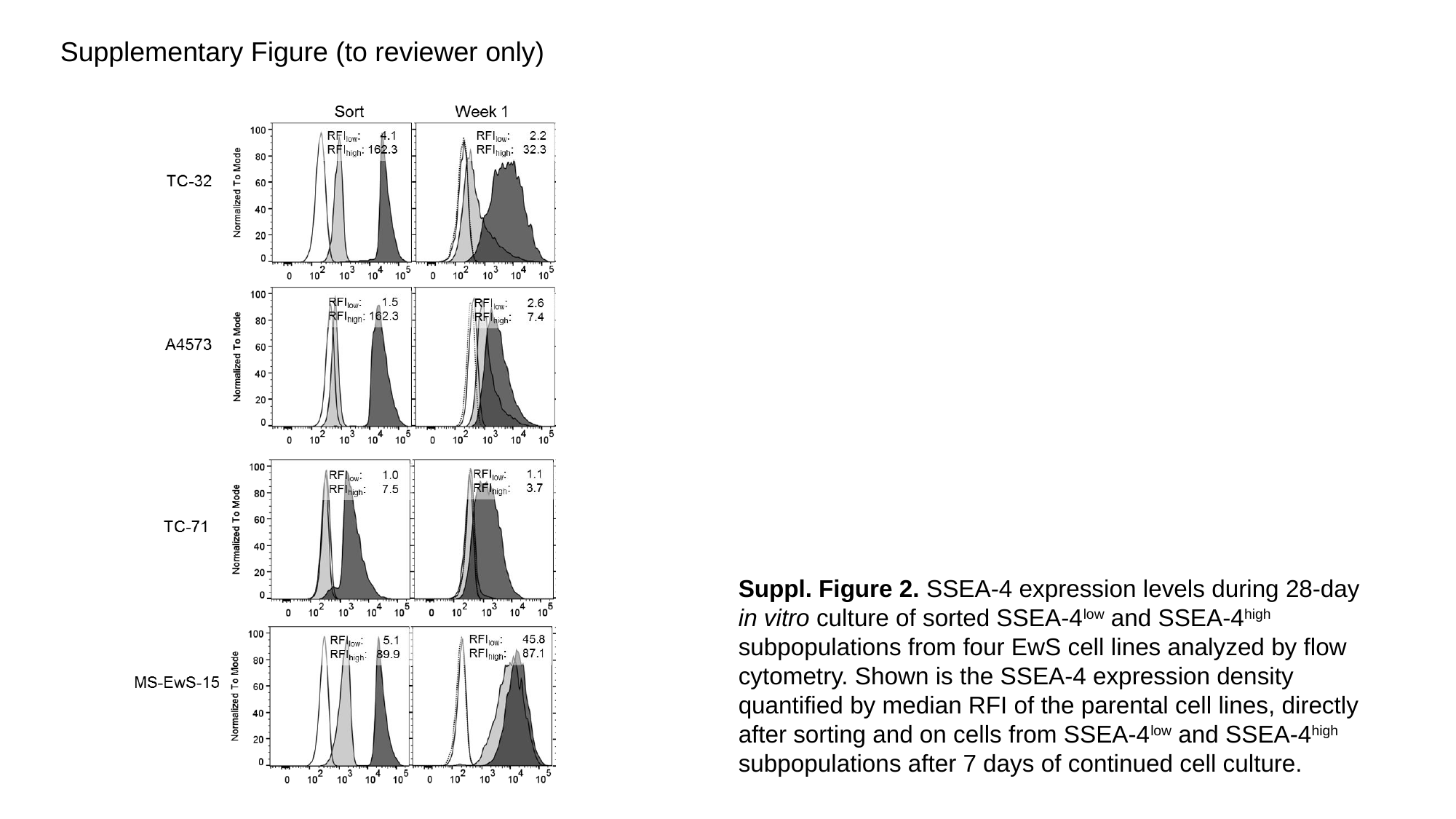

Supplementary Figure (to reviewer only)
Suppl. Figure 2. SSEA-4 expression levels during 28-day in vitro culture of sorted SSEA-4low and SSEA-4high subpopulations from four EwS cell lines analyzed by flow cytometry. Shown is the SSEA-4 expression density quantified by median RFI of the parental cell lines, directly after sorting and on cells from SSEA-4low and SSEA-4high subpopulations after 7 days of continued cell culture.
