# Supplementary material for: Ganglioside SSEA-4 in Ewing sarcoma marks a tumor cell population with aggressive features and is a potential cell-surface immune target
Source: Sci Rep. 2024 May 24;14:11935. doi: 10.1038/s41598-024-62849-8 (PMC11126692; doi:10.1038/s41598-024-62849-8)
Supplement: Supplementary file 3 — Supplementary Table 1. [file 41598_2024_62849_MOESM3_ESM.docx]

**Table 1: Clinical characteristics and SSEA-4 expression in tissue sections**

|  | **Age** | **Gender** | **Fusionprotein** | **Primary tumor site** | **Primary Metastases** | **Status** | **Tissue section** | **Tumor SSEA4** |
| --- | --- | --- | --- | --- | --- | --- | --- | --- |
|  | | | *Tumor tissue sections* | | | | | |
| 1 | 15 | f | EWS-FLI1 | Ankle | P+O+BM | DOD | PT | neg. |
| 2 | 16 | f | EWS-FLI1 | Tibia | no | CR >12 years | PT | neg. |
| 3 | 7 | f | EWS-FLI1 | Pelvis | no | CR1 | PT | pos. + |
| 4 | 15 | m | EWS-FLI1 | Pelvis, Spine | P+B | DOD | PT | pos. ++ |
| 5 | 28 | m | EWS-FLI1 | Pelvis | none | CR1 22 mo | PT | pos ++ |
| 6 | 11 | f | EWS-FLI1 | Tibia | P | DOD | PT | pos.+ |
| 7 | 26 | m | EWS-FLI1 | Pelvis | no | DOD | PT | pos. + |
| 8 | 23 | m | EWS-FLI1 | Humerus | BM, liver | DOD | PT | pos. ++ |
| 9 | 19 | m | EWS-FLI1 | Humerus | P | CR >11years | PT | neg. |
| 10 | 12 | f | EWS-FLI1 | Fibula | no | DOD | PT | pos. + |
| 11 | 11 | m | EWS-FLI1 | Tibia | no | DOD | PT | pos. + |
| 12 | 8 | m | EWS-FLI1 | Chest wall | unknown | CR >6years | PT | neg. |
| 13 | 17 | m | unknown | Tibia | unknown | CR >6years | PT | pos. ++ |
| 14 | 17 | m | unknown | unknown | unknown | DOD | PT | pos. + |
| 15 | 20 | m | EWS-FLI1 | Pelvis | no | DOD | PT | pos. ++ |
| 16 | 20 | m | EWS-FLI1 | Tibia | no | CR1 (9 yrs)** | PT | pos. ++ |
| 17 | 15 | m | unknown | unknown | unknown | unknown | PT | neg. |
| 18 | 32 | f | EWS-FLI1 | Scapula | no | CR >8 years | PT | pos. ++ |
| 19 | 13 | f | EWS-FLI1 | Pelvis | P | DOD | PT | pos. ++ |
| 20 | 17 | f | EWS-FLI1 | Fibula | LK | CR >8 years | PT | pos. ++ |
| 21 | 11 | m | unknown | unknown | unknown | DOD | PT | neg. |
| 22 | 4 | m | unknown | Maxilla | no | unknown | PT | pos. ++ |
| 23 | 18 | f | EWS-FLI1 | Femur | no | CR1 (>9 yrs) | PT | neg. |
| 24 | 19 | f | EWS-FLI1 | Pelvis | no | DOD | PT | pos. ++ |
| 25 | 11 | m | EWS-FLI1 | Vertebral column | P | DOD | M | neg. |
| 26 | 9 | f | EWS-FLI1 | Pelvis | P+B | DOD | M | neg. |
| 27 | 10 | m | unknown | unknown | unknown | unknown | PT | pos. ++ |
| 28 | 19 | m | EWS-FLI1 | Pelvis | P | DOD | M | neg. |
| 29 | 14 | f | unknown | Pelvis | no | DOD | M | pos.+ |
| 30 | 18 | m | unknown | Pelvis | P | DOD | M | pos.+ |
| 31 | 13 | f | EWS-FLI1 | Skull | no | DOD | M | pos.+ |

Abbreviations: BM=bone marrow metastases; CR=complete remission; DOD=dead of disease; f=female; m=male; mo=months; o=osseous metastases; P=pulmonary metastases; PD=progressive disease, PT= Primary Tumor, M= Metastase

All patients had CD99+ small blue round cell tumours diagnosed as Ewing sarcomas by the reference pathology laboratory of the EURO Ewing 99 study center at Gerhard-Domagk-Institute of Pathology in Muenster, and all were treated according to this protocol. SSEA-4 expression was determined by staining with MC 813-70 antibody and subsequent immunofluorescence.
