# Supplementary material for: Ganglioside SSEA-4 in Ewing sarcoma marks a tumor cell population with aggressive features and is a potential cell-surface immune target
Source: Sci Rep. 2024 May 24;14:11935. doi: 10.1038/s41598-024-62849-8 (PMC11126692; doi:10.1038/s41598-024-62849-8)
Supplement: Supplementary file 4 — Supplementary Table 2. [file 41598_2024_62849_MOESM4_ESM.pdf]

Supplementary Table 2. Authentification of cell lines. The identity of the cell lines was confirmed by short tandem repeat (STR) profiling. *http://www.dsmz.de/fp/cgi-bin/str.html

Cell line	D3S1358	vWA	FGA	Ameliogenin	TH01	TPOX	CSF1PO	D5S818	D13S317	D16S539	D7S820	Cell bank profile available?*	
TC-71	15,17	17,18	24,26	x,y	9.30	8,9	10,11	10	11,12	11,14	10	Yes	100% Match
MS-EwS-15	15,17	17,18	20,22	x,y	6.00	8	10,11	11	14	12,13	8,11	No	Unique Profile
MS-EwS-6	14	16,17	20,24	x,y	6.00	8	10,12	13	8,13	8,11	12,13	No	Unique Profile
MS-EwS-16	15,16	16,18	20,26	x,y	6,7	8,10	10,11	13	8,12	11	8,11	No	Unique Profile
TC-32	15,16	15,18	23,24	x	6,9.3	9,11	11,13	12,13	10,12	13,14	8,11	No	Unique Profile
A4573	16,17	18,19	24	x	7.80	8,10	11,13	11,12	8,10	12	11	No	Unique Profile
Cado-ES-1	16,18	14,18	21,22	x	6.90	8,11	11,12	11,12	10,13	9,11	11,13	Yes	100% Match
RD-ES	15	17	21,25	x,y	7.00	9,11	11	11	11,12	9,11	10	Yes	100% Match
5838	16	15,18	20,22	x,y	9.30	8	11	10	13	9,11	11	No	Unique Profile
WE-68	15,18	16,18	19,23	x	6,8	8,10	12	11	9,11	12	10,12	No	Unique Profile
TTC-466	17,18	15,17	24,25	x	7.00	8	10	10	10,12	11,12	8,10	No	Unique Profile
A673	14	15,18	19,20	x	9.30	8	11,12	11,12	8,13	11	10,12	Yes	100% Match
SK-N-MC	15	17,18	21,25	x	9.30	9,11	10	11	11	12	8	Yes	100% Match
VH-64	16,17	15,19	22,23	x	6.00	8	11,12	12,13	8,11	12	12	No	Unique Profile
NTERA-2	16	18,19	23	x,y	9.3	8	10,12	9,12	13	11,12,13	10,12	Yes	100% Match
HT-1080	16	14,19	22,25	x,y	6	8	12,13	11,13	14	9,12	9.1	Yes	100% Match
SUP-B15	15,16	15,17	19,20	x,y	6,9.3	8,9	11,12	12,13	8,14	11,12	10,11	Yes	100% Match

*http://www.dsmz.de/fp/cgi-bin/str.html
